# Rapid and Visual Detection of* Coxiella burnetii* Using Recombinase Polymerase Amplification Combined with Lateral Flow Strips

**DOI:** 10.1155/2018/6417354

**Published:** 2018-04-12

**Authors:** Yong Qi, Qiong Yin, Yinxiu Shao, Suqin Li, Hongxia Chen, Wanpeng Shen, Jixian Rao, Jiameng Li, Xiaoling Li, Yu Sun, Yu Lin, Yi Deng, Wenwen Zeng, Shulong Zheng, Suyun Liu, Yuexi Li

**Affiliations:** ^1^Huadong Research Institute for Medicine and Biotechniques, Nanjing, Jiangsu Province, China; ^2^China Pharmaceutical University, Nanjing, Jiangsu Province, China; ^3^Nanjing Medical University, Nanjing, Jiangsu 210002, China

## Abstract

*Coxiella burnetii*, a global-distributed biological warfare agent, is the causative agent of Q fever. Correct diagnosis of Q fever is challenging and developing a fast, simple, and reliable detection method is necessary. In this study, recombinase polymerase amplification (RPA) assay combined with lateral flow (LF) test was developed targeting 23S rRNA gene of* C. burnetii* Xinqiao strain. Primers and probe were designed and synthesized, with one set with high amplification efficiency screened for establishment of the method. Reaction conditions were optimized. Sensitivity, specificity, and accuracy were evaluated. The established RPA-LF reaction could be completed in 30 minutes by combining RPA at 37°C with LF at room temperature, with visually judged results. The method showed good specificity without recognizing other bacteria evaluated. It detected positive plasmid and genomic DNA at levels of 10 copies/reaction and 7 copies/reaction, respectively, levels comparable to that of real-time quantitative PCR (RT-qPCR) targeting 23S rRNA gene established previously. Both RPA-LF and RT-qPCR were used to detect* C. burnetii*-infected mouse samples and the results were fully consistent. The method showed superior detection performance and will provide technical support against* C. burnetii* in resources-limited areas.

## 1. Introduction

*C. burnetii* is the causative agent of Q fever, which is globally distributed and listed as a biological warfare agent [1]. Its broad host range includes domestic and wild animals as well as humans [2, 3]. Acute Q fever is usually transmitted to humans by inhalation of aerosols generated by infected animals and may progress to chronic disease complicated by endocarditis, chronic hepatitis, and/or osteomyelitis [4, 5], which are sometimes incurable [6, 7].

The largest outbreak of Q fever ever reported in the literature occurred in the Netherlands from 2007 to 2010, and* C. burnetii*-infected dairy sheep or goats were the source [8, 9]. However, accurately diagnosing Q fever is challenging, because its clinical signs, such as flu-like signs or pneumonia, are not pathognomonic [10, 11], leading to misdiagnosis, delayed treatment, serious pneumonia, and even fatal disease [12]. To reducing such major impacts of Q fever on public health, the development of accurate diagnostic techniques is firstly required [13].

Specialized laboratories detect Q fever by diagnostic techniques employing antibodies or nucleic acid. The most commonly used and reliable antibody detection methods include complement fixation [14], indirect immunofluorescence [15], and ELISA [16]. These methods rely on antibodies generating usually in 1 to 2 weeks after infection, which may delay proper treatment. Also preparing the diagnostic* C. burnetii* whole cell antigens is hazardous and laborious [3, 13, 17]. The culture of the antigen usually needs a biological safety protection third-level laboratory, where lots of human and material resources are needed, or there is a risk of leakage to threat public health. As a nucleic acid detection method, real-time quantitative PCR (RT-qPCR) is the most frequently used for direct detection of* C. burnetii* in whole blood or buffy coat aliquots collected at onset of symptoms and before antibiotic treatment [18, 19]. This method relies on highly equipped centralized laboratories as the fluorescent quantitative machine is too expensive, especially in resource-limited areas where these diseases are endemic.

It appeared, thus, necessary to develop a fast, simple, and reliable detection method for* C. burnetii*, which is suitable for diagnosis in the field, in simple and crude removable laboratory, or in some basic medical unit such as county or township hospital. Recombinase polymerase amplification (RPA) is an isothermal DNA amplification method in which amplification reaction can be completed in 10 to 20 min at 24°C to 45°C [20]. The successful application of RPA is evident as shown in many publications [21]. Furthermore, the amplicons can be visually detected using a lateral flow (LF) test at room temperature. A viable point-of-care nucleic acid detection method combining RPA with LF is promising for utilization in resources-limited areas. In this study, such a detection method for* C. burnetii* was established and evaluated.

## 2. Materials and Methods

### 2.1. Ethics Statement

The animal experiments were approved by the Administrative Committee for Laboratory Animals of Huadong Research Institute for Medicine and Biotechniques and the animal care met the standard of the committee. Mice were well cared for during their stay in the facility and all efforts were made to minimize suffering. The use of human blood samples was approved by the Ethics Committee of Huadong Research Institute for Medicine and Biotechniques and consent form was signed.

### 2.2. Preparations of DNA

DNA from spleens of* C. burnetii *(Xinqiao strain)-infected mice was kindly given by Professor Bohai Wen from State Key Laboratory of Pathogens and Biosafety of China. Briefly, 9 female, 5-week old C57BL/6 mice were infected with* C. burnetii* through intraperitoneal injection, and 9 control mice were injected with phosphate buffered saline (PBS). One week after infection, mice were sacrificed and spleens were collected. DNA was extracted from 10 mg of the spleens using a QIAamp Blood and Tissue Mini DNA kit (Qiagen, CA, USA) as per the manufacturer's instruction.

Genomic DNAs of* C. burnetii *(Xinqiao strain),* Rickettsia rickettsii *(Sheila Smith),* Rickettsia heilongjiangensis *(054 strain), and* Rickettsia sibirica* (246 strain) were also kindly given by Professor Wen. Bacteria of* Orientia tsutsugamushi *(Karp-like strain),* Staphylococcus aureus*, and* Streptococcus suis* were kindly given by Research Institute for Medicine of Nanjing Command [23–25]. Genomic DNAs of these bacteria were extracted from the corresponding bacteria using a QIAamp Blood and Tissue Mini DNA kit (Qiagen).

For human blood DNA, 5 mL of blood samples was collected from cubital veins of health volunteers and DNA was extracted using a QIAamp Blood and Tissue Mini DNA kit (Qiagen).

For quality control, the presence of DNAs of* C. burnetii*,* R. rickettsii*,* R. heilongjiangensis*,* R. sibirica*,* O. tsutsugamushi*,* S. aureus*, and* S. suis* was detected using RT-qPCR as described previously [22, 26–31] and that of human blood was detected using PCR targeting GAPDH gene [32].

### 2.3. Design of Primers and Probes

The sequence of 23S rRNA gene of* C. burnetii* (Genbank: AE016828) was analyzed using DNAman version 5.2.2 software and Nucleotide BLAST online (https://blast.ncbi.nlm.nih.gov/Blast.cgi). Partial sequence of the 23S rRNA gene, from 169923 to 170964 of the genome sequence, was selected as target gene and its specificity was evaluated by Nucleotide BLAST online. Polymerase chain reaction (PCR) primers were designed using software Primer premier 5.0 to include restriction enzyme cutting sites of* Bam*H I or* Eco*R I as shown in Table 1. The primers and probe for RPA were designed manually as indicated in Table 1, including 4 forward primers, 4 reverse primers, and 1 probe. The 5′ end of the reverse primer was labeled with biotin. The 5′ end of the probe was labeled with Carboxyfluorescein (FAM), the 3′ end was blocked with a phosphate group, and a base analog tetrahydrofuran (THF) was inserted between the 30th and 31st base. All primers and probes were synthesized by Genscript company of Nanjing.

### 2.4. Construction of Recombinant Plasmid

The partial gene of 23S rRNA was amplified by PCR using primers indicated above. Briefly, 12.5 *μ*L of 2x PCR premix solution (Premix Taq™ Version 2.0, TaKaRa, Dalian, China), 1.5 *μ*L of each primer (10 nM), 2 *μ*L of genomic DNA (1 × 10^8^ copies/*μ*L), and 7.5 *μ*L of dH2O were mixed together to initiate the reaction. The reaction was conducted as described by the manufacturer's instruction. Plasmid pUC19 was purified from* E. coli* cells using a TaKaRa MiniBEST Plasmid Purification Kit. Both the amplified gene and plasmid were digested using* BamH* I and* Eco*R I restriction enzymes and linked using a Takara DNA Ligation Kit. Competent* E. coli* cells were transformed with ligation product, scraped to solid LB medium with ampicillin, and incubated overnight at 37°C. Recombinant plasmid 23SrRNA-pUC19 from positive bacterial colony was purified and digested using both* BamH* I and* Eco*R I, and the product was analyzed by agarose gel electrophoresis to confirm the target gene was linked. The concentration of the recombinant plasmid was measured using Nanodrop 2000, and the copies were calculated according to Avogadro constant.

### 2.5. Establishment and Optimization of RPA Assay

For establishment of the RPA method, each of the 4 RPA forward primers was combined with each of the 4 RPA reverse primers to make 16 groups of primers. An initial RPA reaction system recommended by the manufacturer's instruction of TwistAmp® RPA nfo kit (TwistDx™ Limited, Cambridge, UK) was used to screen the best primer group. Briefly, 29.5 *μ*L of rehydration buffer, 2.1 *μ*L of forward or reverse primer (10 *μ*M), 0.6 *μ*L of probe (10 *μ*M), 1 *μ*L of 23SrRNA-pUC19 or pUC19 (1 × 10^4^ copies/*μ*L), and 12.2 *μ*L of dH_2_O were mixed together, vortexed, and spun briefly. The mixture was then added to the 0.2 mL tube which contained the freeze-dried reaction pellet to reconstitute it. The reaction pellet consisted of the recombinase, polymerase, and single-stranded binding protein. 2.5 *μ*L of magnesium acetate (280 mM) was pipetted into the tube lids. To initiate the reaction, the lids were closed and the magnesium acetate was spun down using a microcentrifuge mySPIN 6 (Thermo Scientific) for 5 sec to the mixture. The tube was incubated at 37°C for 4 min, followed by another brief vortex and incubation for 16 min at 37°C.

For analysis of the amplified product, Millenia Genline Hybridetect-1 (MGH) strips (Millenia Biotec GmbH, Gieben, Germany) were used. Two *μ*L of the amplified products were mixed with Tris-buffered saline to a total of 100 *μ*L in a well of a 96-well plate. The sample pad of each MGH strip was immersed into the dilution of amplified product in each well. After 3 to 5 min of incubation at room temperature, the results were determined visually by naked eyes from the test line (T line) and the control line (C line) on the strips. Briefly, as a positive result, both the T line and the C line were developed, indicating that the DNA labeled with both FAM and biotin existed in the amplicons; only the C line being developed indicates a negative result; only the T line being developed indicates that the strip should be replaced.

To optimize the RPA-LF system, various concentrations of reverse primer (10 *μ*M, 5 *μ*M, and 2.5 *μ*M) and probe (5 *μ*M and 2.5 *μ*M) were evaluated in the reaction mixture, a series of amplification times were explored, and various volumes of amplified products (1 *μ*L, 2 *μ*L, and 5 *μ*L) were used to develop the MGH strips. In the optimization experiment, 1 × 10^4^ copies of 23SrRNA-pUC19 (experimental group) or pUC19 plasmid (negative control) was used as template. All the reactions were conducted in duplication.

### 2.6. Evaluation of Sensitivity and Specificity

Both recombinant plasmid 23SrRNA-pUC19 and genomic DNA of* C. burnetii* were used to evaluate the sensitivity of the established RPA-LF detection method.

The recombinant plasmid was diluted into a series of concentrations from 1 × 10^4^ copies/*μ*L to 1 copy/*μ*L with Elution buffer (TaKaRa MiniBEST Plasmid Purification Kit Ver.4.0) and added to the optimized RPA reaction system as templates to evaluate the detection limit of the method for detection of positive plasmid. The genomic DNA of* C. burnetii* was diluted into a series of dilutions with human blood DNA solution and the DNA copies were evaluated using RT-qPCR as described previously [5, 22]. Primers and probe sequences used in RT-qPCR were indicated in Table 1. Then these dilutions were used as templates to evaluate the detection limit of the method for detection of genomic DNA.

For evaluation of specificity, the purified genomic DNAs of* C. burnetii*,* R. rickettsii*,* R. heilongjiangensis*,* R. sibirica*,* O. tsutsugamushi*,* S. aureus*, and* S. suis* were used as experimental or control templates to conduct the RPA detection method.

All the reactions were conducted in duplication.

### 2.7. Detection of Infected Mouse Samples

DNA from infected or uninfected mouse spleens was prepared as described above.* C. burnetii* DNA in the samples was detected using RT-qPCR targeting 23S rRNA gene as described previously [5, 22] which was considered as the golden standard in this research. The primers and probe used in RT-qPCR were indicated in Table 1. Then the samples were used as templates to evaluate the established RPA detection method. The results of RPA were compared with those of RT-qPCR to check their accuracy.

## 3. Results

### 3.1. Specific Sequence and Recombinant Plasmid

**Figure 1:**
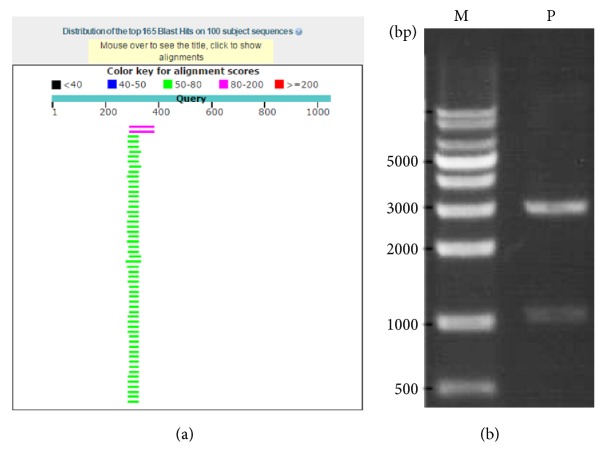
Specificity analysis of the partial sequence of 23S rRNA gene and verification of the recombinant plasmid. (a) The partial sequence was analyzed using Nucleotide BLAST with genome of* C. burnetii* excluded and the BLAST hits were shown; (b) the recombinant plasmid was digested with* Bam*H I and* Eco*R I, and the products were analyzed using agarose gel electrophoresis; M, DNA marker; P, digested products.

Partial sequence of 23S rRNA gene of* C. burnetii* was selected as a specific sequence. Its specificity was evaluated using Nucleotide BLAST online with* C. burnetii* (taxid: 777) excluded in the interface of the software. As shown in Figure 1(a), the partial sequence selected was very specific and the alignment scores were no higher than 200 aligned with DNA sequence of any other species. This ensured the “in silico” specificity of the RPA assay.

The DNA sequence was linked with pUC19 plasmid to make a recombinant plasmid, which was verified by digestion with* Bam*H I and* Eco*R I and analyzed with agarose gel electrophoresis as shown in Figure 1(b). The band on the gel corresponded to the actual size of the DNA sequence of 1042 bp, indicating the successful construction of recombinant plasmid.

### 3.2. RPA Assay Establishment and Optimization

Four forward primers, 4 reverse primers, and 1 probe were designed, synthesized, and combined to make 16 primer groups. The best primer group was screened using the initial RPA reaction system. A best primer group should not only lead to a high amplification efficacy, but also lead to a good specificity. So except for the recombinant 23SrRNA-pUC19 as the experimental template, an empty plasmid pUC19 was used as the control template. After the amplification, the products were developed with lateral flow detection strips and visually determined the results [21].

**Figure 2:**
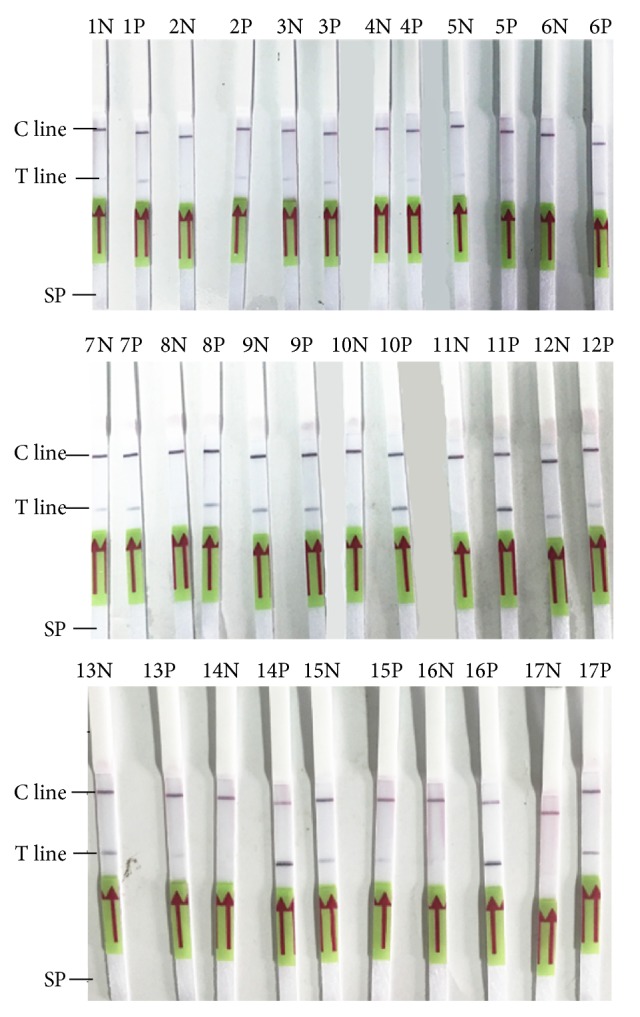
Screening of primer combinations. Sixteen groups of primers (indicated in the table) were used to conduct the RPA-LF reaction with recombinant plasmid 23SrRNA-pUC19 as positive template (P) and plasmid pUC19 as negative template (N). Group 17 using primers, probe, and template from the TwistAmp RPA nfo kit was conducted as a control. SP, sample pad of the strip.

As shown in Figure 2 and Table 2, T lines of the experimental strips in groups 10, 11, 14, and 16 developed band with deeper color while these of the control strips developed no band. These four groups of primers performed best amplification efficacy and specificity. In the other groups, T lines of the experimental strips developed lighter color band, or T lines of both experimental and control strips developed band, indicating a low amplification efficacy or poor specificity. The primer group 16 was selected for the experiment as shown in Table 2.

**Figure 3:**
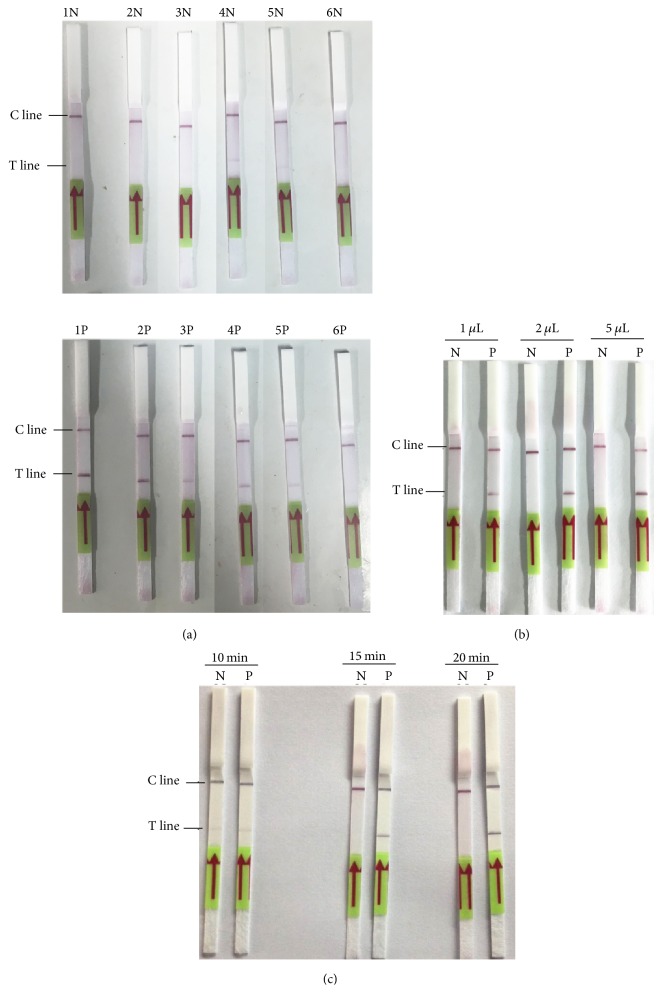
Optimization of the RPA-LF detection system. (a) Various concentrations of reverse primer and probe were combined to make 6 groups as indicated in Table 3 and evaluated using RPA-LF; (b) various volumes (1 *μ*L, 2 *μ*L, or 5 *μ*L) of the amplified products were used to develop the MGH strips; (c) various of amplification times (10, 15, or 20 min) were used in RPA-LF and developed with MGH strips. Each group used both recombinant plasmid 23SrRNA-pUC19 as positive template (P) and plasmid pUC19 as negative template (N).

The RPA-LF detection system was optimized using primer group 16. Different concentrations of reverse primer and probe were combined and introduced to the RPA assay. As shown in Figure 3(a) and Table 3, T lines of the experimental strip in groups 1 (10 *μ*M of CbR564 and 5 *μ*M of Cbprobe408) and 2 (5 *μ*M of CbR564 and 5 *μ*M of Cbprobe408) developed band with deeper color while those of the control strip developed no band. Considering the material cost, a lower concentration of reverse primer was selected with 5 *μ*M of CbR564 and 5 *μ*M of Cbprobe408. Various loading volumes of the amplified products were used to develop the MGH strips. As shown in Figure 3(b), the color intensities of the bands were proportional to the loading volumes and 5 *μ*L of the amplified products developed the deepest band on the strip. Various amplification times were set for RPA assay to determine the most proper time. As shown in Figure 3(c), an amplification time of 15 min could lead to a modest development on the strip and 20 min to the deepest band. Considering a better sensitivity, a loading volume of 5 *μ*L of amplified product and an amplification time of 20 min were chosen in the optimized RPA-LF detection method.

### 3.3. Sensitivity and Specificity

**Figure 4:**
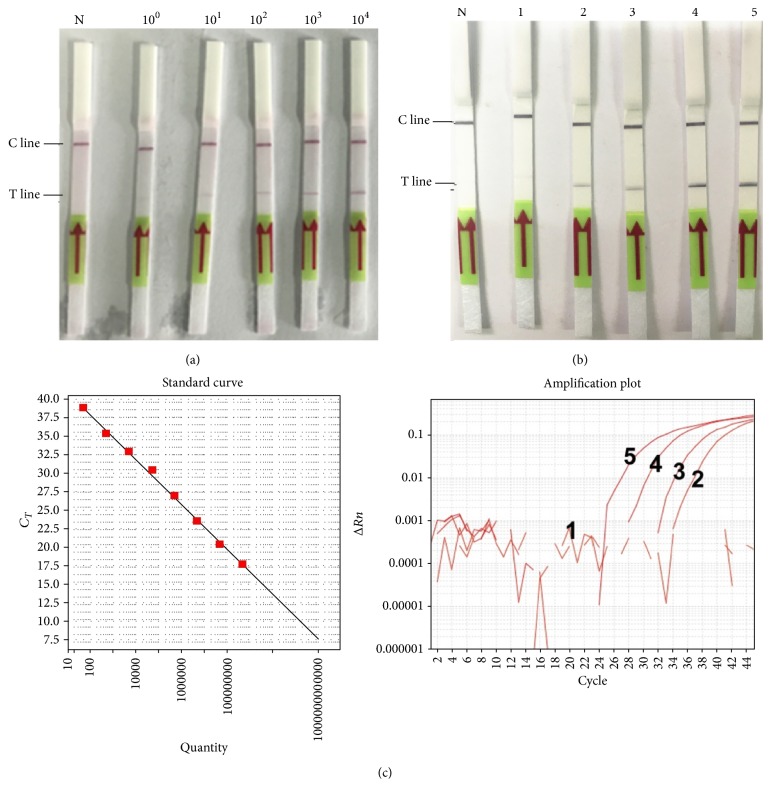
Sensitivity analysis of RPA-LF detection method. Sensitivity of RPA-LF detection method was evaluated with various copies of 23SrRNA-pUC19 (a) or various of dilutions of genomic DNA of* C. burnetii *(b). Copies of genomic DNA of dilutions 1 to 5 (as shown on (b)) were evaluated as undetected, 7 copies/*μ*L, 51 copies/*μ*L, 1002 copies/*μ*L, and 14526 copies/*μ*L, respectively, by RT-qPCR (c). N, negative control using template of pUC19 plasmid (a) or human blood DNA (b).

The sensitivity of the RPA-LF detection method in detecting recombinant plasmid 23SrRNA-pUC19 or genomic DNA of* C. burnetii* was evaluated. As shown in Figure 4(a), 10 to 10,000 copies of recombinant 23SrRNA-pUC19 could be detected in the reaction while fewer copies could not. The detection limit of the method is 10 copies/reaction in detecting recombinant plasmid. For genomic DNA, 5 dilutions were tested with both the established RPA-LF method and RT-qPCR. After the RPA reaction, Genomic DNA of* C. burnetii* in dilutions 2 to 5 could be detected while that in dilution 1 was not detected (Figure 4(b)). In the RT-qPCR, genomic DNA copies in dilutions 2 to 5 were detected as 7 copies/*μ*L, 51 copies/*μ*L, 1002 copies/*μ*L, and 14526 copies/*μ*L, respectively, while those in dilution 1 were undetected (Figure 4(c)). Compared with RT-qPCR, the detection limit of RPA-LF method in detecting genomic DNA could be as low as 7 copies/reaction, which is similar to that of RT-qPCR.

**Figure 5:**
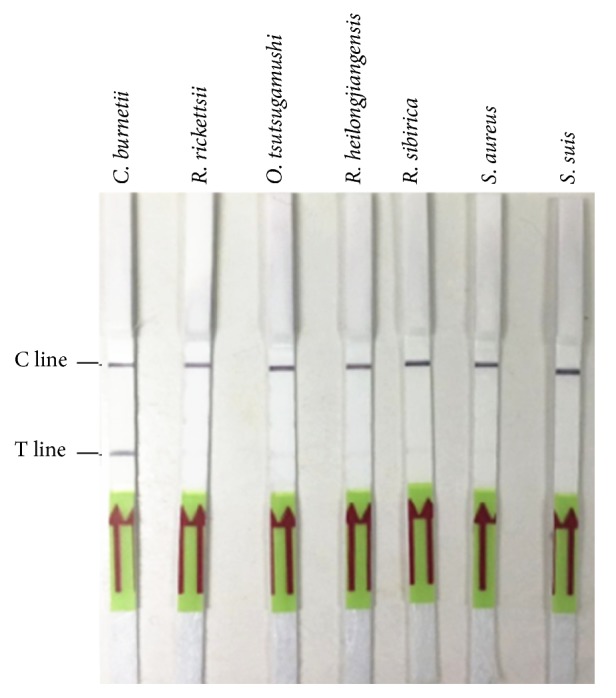
Specificity analysis of RPA-LF detection method. Genomic DNA from various organisms including* C. burnetii*,* R. rickettsii*,* R. heilongjiangensis*,* R. sibirica*,* O. tsutsugamushi*,* S. aureus*, and* S. suis* was used as experimental or control templates to conduct the RPA-LF detection method.

Except for genomic DNA of* C. burnetii*, genomic DNAs of other pathogens, including* R. rickettsii*,* R. heilongjiangensis*,* R. sibirica*,* O. tsutsugamushi*,* S. aureus*, and* S. suis* were used to evaluate the specificity of the RPA-LF method. The concentrations of these genomic DNAs were determined to be 1 × 10^5^ to 1 × 10^8^ copies/*μ*L by RT-qPCR. As a result shown in Figure 5, the established method showed no cross-reaction with the genomic DNAs of these 6 kinds of pathogens and performed a good specificity.

### 3.4. Infected Mouse Samples Detection

**Figure 6:**
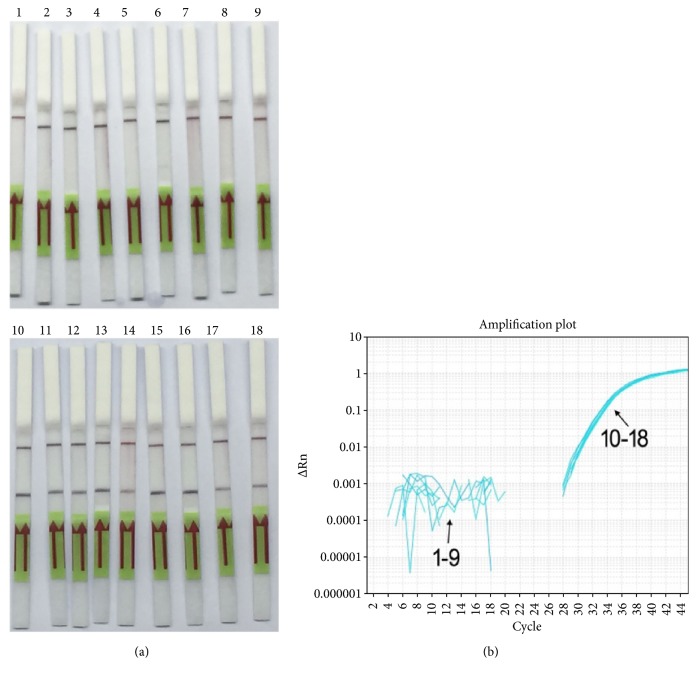
Evaluation of the established RPA-LF method with* C. burnetii*-infected animal samples. DNAs of uninfected (1 to 9) or* C. burnetii*-infected (10 to 18) mouse spleens were detected with RPA-LF method (a) or RT-qPCR (b).

The effectiveness of the established RPA-LF method was evaluated with DNA of spleens from* C. burnetii*-infected mice. The existence of* C. burnetii* DNA was verified by RT-qPCR. As shown on Figure 6,* C. burnetii* DNA in the 9 samples was all detected while that in the 9 control samples was not. The results showed a good coincidence between RPA-LF method and RT-qPCR. The established method is effective in detection of infected mouse samples with both sensitivity and specificity of 100%.

## 4. Discussion

RPA is a new developed technology and is considered as a replacement of PCR in the future [33]. It mainly relies on three proteins, a recombinase, a polymerase, and a single strand binding protein. The optimum reaction temperature of these proteins is 37°C. In the reaction process, constant change of temperature like PCR or RT-qPCR was not necessary, resulting in a highly efficient amplification and consuming less than 20 min. However, until now, no reliable method of primer or probe design for RPA exists and optimal sets of primers and probes need to be screened. In this research, 4 of the 16 primer groups were screened to lead to a high amplification efficacy and a modest specificity. In the optimization, though we chose an amplification time of 20 min for a better sensitivity, the 15 min of amplification did lead to a deep enough band on the strip. In the actual clinical detection application, the amplification time can be set from 15 to 20 min.

In this study, partial sequence of 23S rRNA gene of reference strain RSA 493 was chosen and finally a sequence of 166 bp between primers CbF399 and CbR564 was selected as target sequence. To ensure that the established method could detect all the other strains of* C. burnetii*, the target sequence was aligned with the corresponding sequence of almost all the strains in Genbank database using Nucleotide BLAST online (https://blast.ncbi.nlm.nih.gov/Blast.cgi), including strains Schperling (Genbank: CP014563.1), Heizberg (Genbank: CP014561.1), Henzerling (Genbank: CP014559.1), 18430 (Genbank: CP014557.1), 2574 (Genbank: CP014555.1), 701CbB1 (Genbank: CP014553.1), 14160-001 (Genbank: CP014551.1), 14160-002 (Genbank: CP014836.1), CbCVIC1 (Genbank: CP014549.1), 42785537 (Genbank: CP014548.1), Scurry_Q217 (Genbank: CP014565.1), RSA 439 (Genbank: CP018005.1 and CP020616.1), MSU Goat Q177 (Genbank: CP018150.1), RSA 493 (Genbank: AE016828.3), 3345937 (Genbank: CP014354.1), 3262 (Genbank: CP013667.1), Cb175_Guyana (Genbank: HG825990.3), Namibia (Genbank: CP007555.1), Z3055 (Genbank: LK937696.1), CbuK_Q154 (Genbank: CP001020.1), CbuG_Q212 (Genbank: CP001019.1), RSA 331 (Genbank: CP000890.1), and Dugway 5J108-111 (Genbank: CP000733.1). All the sequences from various strains are of 100% identity, indicating that the established method could detect almost all* C. burnetii *strains theoretically.

The amplicons of RPA assay can be detected using agarose gel electrophoresis, lateral flow test, or real-time fluorescent detection. Both the agarose gel electrophoresis and real-time fluorescent detection need expensive devices or are not convenient enough for basic medical unit like some county or township hospitals. So we chose lateral flow test for the product detection in which results can be visually determined. To do this, a nfo (a kind of endonuclease) enzyme, a biotin-labeled reverse primer, and a FAM-labeled, phosphate-blocked, and THF-inserted probe were introduced. After a normal RPA process, the amplified products will be labeled with biotin. Then the probe will be annealed with the amplicons, and the blocked 3′ end of the probe will be cut off by nfo enzyme at the THF site. The left probe sequence labeled with FAM will initiate another amplification as a forward primer with the biotin-labeled reverse primer, resulting in a double labeled product, which will be recognized by the MGH strips. The design by the company is artful; however, the role that the forward primer plays is limited and we suspect that even if the forward primer is removed, the whole reaction still works. This will be tested and discussed in the future. During the research, it is found that the amplified products were easy to spread when pipetted to develop the strips, leading to a potentially important contamination and false positive in the future experiment. So maybe using a XCP cassette to replace the MGH strips is a good option as suggested by Chao et al. [21].

The RPA assay has been shown to detect product amplified from a single molecule [20, 21]. This research shows that RPA is a method that could be used to detect plasmid DNA, DNA extracted from* C. burnetii*-infected mice, or pure organisms with a detection limit of 10 copies or less, within 20 minutes, and without cross-reaction with other organisms. This is generally comparable to that of RT-qPCR considering they have a similar detection limit (Figure 4). Actually, RPA is more attractive. A cheap heating block is the only needed device instead of an expensive fluorescent quantitative machine in RT-qPCR, which liberates some detection limits for many different laboratories with limited instruments and infrastructure.

In the specificity analysis, 4 kinds of bacteria (*R. rickettsii*,* R. heilongjiangensis*,* R. sibirica*, and* O. tsutsugamushi*) from order of Rickettsiales were chosen as relative control for* C. burnetii* shares similar biological characteristics and was classified into the order of Rickettsiales in the past. Also two irrelevant kinds of bacteria (*S. aureus* and* S. sui*s) were used to exclude potential influence or interference from other disease. The established method did not recognize any of these bacteria, indicating a good specificity of this method.

In this research, it is a pity that we did not collect any clinical samples from patients. So samples from* C. burnetii*-infected mice were used to evaluate the established method. The results totally agree with those of RT-qPCR. For acute human cases, whole blood or buffy coat aliquots are most useful for diagnosis [19]. So in the sensitivity evaluation experiment, the genomic DNA was diluted with human blood DNA solution, which partly simulated the patient samples and excluded the interference of human DNA in the method. All the results demonstrated the potential clinical application of the assay.

In conclusion, based on RPA assay and LF strips, we successfully establish a prompt, accurate, sensitive, specific method for detection of* C. burnetii*, with a visible result judged by naked eyes. The method had a detection limit similar to that of RT-qPCR and a modest specificity without recognizing other organisms like* R. rickettsii*,* R. heilongjiangensis*,* R. sibirica*,* O. tsutsugamushi*,* S. aureus*, or* S. suis*. and samples from mouse or human. The results evaluated with infected mouse samples were in complete agreement with those of RT-qPCR. The isothermal amplification, at a constant temperature of 37°C, obtained by RPA-LF makes the method promising for a wide use in the field, though more clinical patient samples are needed to evaluate the method in the future.

## Figures and Tables

**Table 1:** Primers and probe for PCR, RPA, and qPCR.

Usages	Primer or probe	Nucleotide sequences (5′-3′)
PCR	CbF1	AAGGATCCAATTAACCGTTGTAGTT
	CbR1042	CGGAATTCTCACTCTTTCCTATGTT

RPA	CbF310	TCCTTGTCGGGTAAAAAATTGCCCCGCTAA
	CbF340	ACTGTAAAGTTTAGTGATAAAGTCAGCTCA
	CbF370	TATCGGGGGAACCCTCCTGCTTTTTAGCAA
	CbF399	AAGGGCAATCCCGAGGGAAGTCTTAAATGA
	CbR484	Biotin-AGTCAGCGTATTGCACACAAATGCGTGCCT
	CbR507	Biotin-TGAGTATAAACCCAAGGGCAAGAAGTCAGC
	CbR533	Biotin-TAAATTCTCCATAGTCACTTACTTCTTGAG
	CbR564	Biotin-CATACCATGGCTCTAAATGTAAATACATAA
	Cbprobe408	FAM-CCCGAGGGAAGTCTTAAATGACCCCGTAAC-[THF]-ACTGATCCGAAAGGT-PO_4_

RT-qPCR [22]	CbF	CGGCTGAATTTAAGCGATTTATTTTT
	CbR	CGTAACCACACACGCATCTCA
	TaqMan-MGB probe	TGCAATGGGTTCGG

**Table 2:** Results of screening of primer combinations.

Group number	Primer pairs	Results
1	CbF310 + CbR484	−
2	CbF310 + CbR507	−
3	CbF310 + CbR533	−
4	CbF310 + CbR564	−
5	CbF340 + CbR484	−
6	CbF340 + CbR507	−
7	CbF340 + CbR533	−
8	CbF340 + CbR564	+
9	CbF370 + CbR484	−
10	CbF370 + CbR507	+
11	CbF370 + CbR533	+++
12	CbF370 + CbR564	−
13	CbF399 + CbR484	−
14	CbF399 + CbR507	+++
15	CbF399 + CbR533	−
16	CbF399 + CbR564	+++

^−^Bad; ^+^good; ^+++^excellent.

**Table 3:** Results of RPA-LF using reverse primer and probe with various concentrations.

Group number	Concentrations (CbR564 & Cbprobe408)	Results
1	10 *μ*M & 5 *μ*M	+++
2	5 *μ*M & 5 *μ*M	+++
3	2.5 *μ*M & 5 *μ*M	+
4	10 *μ*M & 2.5 *μ*M	−
5	5 *μ*M & 2.5 *μ*M	−
6	2.5 *μ*M & 2.5 *μ*M	−

^−^Bad; ^+^good; ^+++^excellent.
